# BGmisc: An R Package for Extended Behavior Genetics Analysis

**DOI:** 10.21105/joss.06203

**Published:** 2024-02-24

**Authors:** S. Mason Garrison, Michael D. Hunter, Xuanyu Lyu, Jonathan D. Trattner, S. Alexandra Burt

**Affiliations:** 1 Department of Psychology, Wake Forest University, North Carolina, USA; 2 Department of Human Development and Family Studies, Pennsylvania State University, Pennsylvania, USA; 3 Institute for Behavioral Genetics, University of Colorado at Boulder, Colorado, USA; 4 Department of Psychology & Neuroscience, University of Colorado at Boulder, Colorado, USA; 5 Department of Psychology, Michigan State University, Michigan, USA

## Abstract

Behavior genetics is a field that studies how our genes and environment contribute to differences in behavior and traits among individuals. Traditionally, twin studies have long been a cornerstone of this field, helping researchers understand how genetics influence behavior. Recently, the focus has expanded to include studies with more complex family structures, e.g., children of twins (CoT, D’Onofrio et al., 2003) and mother-daughter-aunt-niece (MDAN, Rodgers, Bard, Johnson, D’Onofrio, & Miller, 2008). These broader studies offer more detailed insights into how our genes and environment shape us, but they also make analyzing and organizing the data more complex. BGmisc simplifies the analysis of these complex data structures by offering a comprehensive suite of functions for accommodating families of any size, from twins to extensive pedigrees.

## Statement of need

The move towards analyzing complex family structures in behavior genetics introduces challenges in data structuring and modeling. The data structures inherent in these more complicated family designs are orders of magnitude larger than traditional designs. For example, in the classical twin study, a family will consist of a single pair of twins (i.e., two people), whereas in the MDAN design, a family consists of two mother-daughter pairs (i.e., four people). This problem quickly becomes intractable when applied to extended family pedigrees, which can encompass up to hundreds of thousands of individuals in a single family (e.g, Garrison et al., 2023).

This shift towards extended family models underscores the limitations of existing genetic modeling software. Packages like OpenMx (Neale et al., 2016), EasyMx (Hunter, 2023), and kinship2 (J. P. Sinnwell, Therneau, & Schaid, 2014; J. Sinnwell & Therneau, 2022) were developed with smaller, classical family designs in mind. In contrast, the BGmisc R package was specifically developed to structure and model extended family pedigree data.

Two widely-used R packages in genetic modeling are OpenMx (Neale et al., 2016) and kinship2 (J. P. Sinnwell et al., 2014; J. Sinnwell & Therneau, 2022). The OpenMx (Neale et al., 2016) package is a general-purpose software for structural equation modeling that is popular among behavior geneticists (Garrison, 2018) for its unique features, like the mxCheckIdentification() function. This function checks whether a model is identified, determining if there is a unique solution to estimate the model’s parameters based on the observed data. In addition, EasyMx (Hunter, 2023) is a more user-friendly package that streamlines the process of building and estimating structural equation models. It seamlessly integrates with OpenMx’s infrastructure. Its functionalities range from foundational matrix builders like emxCholeskyVariance and emxGeneticFactorVariance to more specialized functions like emxTwinModel designed for classical twin models. Despite their strengths, EasyMx and OpenMx have limitations when handling extended family data. Notably, they lack functions for handling modern molecular designs (Kirkpatrick, Pritikin, Hunter, & Neale, 2021), modeling complex genetic relationships, inferring relatedness, and simulating pedigrees.

Although not a staple in behavior genetics, the kinship2 (J. P. Sinnwell et al., 2014) package provides core features to the broader statistical genetics scientific community, such as plotting pedigrees and computing genetic relatedness matrices. It uses the Lange algorithm (Lange, 2002) to compute relatedness coefficients. This recursive algorithm is discussed in great detail elsewhere, laying out several boundary conditions and recurrence rules. The BGmisc package extends the capabilities of kinship2 by introducing an alternative algorithm to calculate the relatedness coefficient based on network models. By applying classic path-tracing rules to the entire network, this new method is computationally more efficient by eliminating the need for a multi-step recursive approach.

## Features

The BGmisc package offers features tailored for extended behavior genetics analysis. These features are grouped under two main categories, mirroring the structure presented in our vignettes.

### Modeling and Relatedness

- Model Identification: BGmisc evaluates whether a variance components model is identified and fits the model’s estimated variance components to observed covariance data. The technical aspects related to model identification have been described by Hunter, Garrison, Burt, & Rodgers (2021).
- Relatedness Coefficient Calculation: Using path tracing rules first described by Wright (1922) and formalized by McArdle & McDonald (1984), BGmisc calculates the (sparse) relatedness coefficients between all pairs of individuals in extended pedigrees based solely on mother and father identifiers.
- Relatedness Inference: BGmisc infers the relatedness between two groups based on their observed total correlation, given additive genetic and shared environmental parameters.

### Pedigree Analysis and Simulation

- Pedigree Conversion: BGmisc converts pedigrees into various relatedness matrices, including additive genetics, mitochondrial, common nuclear, and extended environmental relatedness matrices.
- Pedigree Simulation: BGmisc simulates pedigrees based on parameters including the number of children per mate, generations, sex ratio of newborns, and mating rate.

Collectively, these tools provide a valuable resource for behavior geneticists and others who work with extended family data. They were developed as part of a grant and have been used in several ongoing projects (Burt, 2023; Garrison et al., 2023; Hunter et al., 2023; Lyu et al., 2023) and theses (Lyu, 2023).
